# Can the COVID-19 Pandemic Disrupt the Current Drug Development Practices?

**DOI:** 10.3390/ijms22115457

**Published:** 2021-05-21

**Authors:** Jung-Hyun Won, Howard Lee

**Affiliations:** 1 Department of Molecular Medicine and Biopharmaceutical Sciences, Graduate School of Convergence Science and Technology, Seoul National University, Seoul 03080, Korea; jhwon@snu.ac.kr; 2 Center for Convergence Approaches in Drug Development, Graduate School of Convergence Science and Technology, Seoul National University, Seoul 03080, Korea; 3 Department of Clinical Pharmacology and Therapeutics, Seoul National University College of Medicine, Seoul 03080, Korea; 4 Department of Clinical Pharmacology and Therapeutics, Seoul National University Hospital, Seoul 03080, Korea; 5 Department of Transdisciplinary Studies, Graduate School of Convergence Science and Technology, Seoul National University, Seoul 03080, Korea; 6 Advanced Institute of Convergence Technology, Suwon 16229, Korea

**Keywords:** COVID-19, drug development, paradigm shift

## Abstract

Therapeutics and vaccines against the COVID-19 pandemic need to be developed rapidly and efficiently, given its severity. To maximize the efficiency and productivity of drug development, the world has adopted disruptive technologies and approaches in various drug development areas. Telehealth, characterized by the heavy use of digital technologies; drug repositioning strategies, aided by computational breakthroughs; and data tracking tool hubs, enabling real-time information sharing, have received much attention. Moreover, drug developers have engaged in open innovation by establishing various types of collaborations, many of which have been carried out across nations and enterprises. Finally, regulatory agencies have attempted to operate on a more flexible review basis than before. Although such disruptive approaches have partly reshaped drug development practices, issues and challenges remain before the completion of this paradigm shift in conventional drug development practices for the post-pandemic era. In this review, we have highlighted the role of a collaborative community of experts in order to figure out how disruptive technologies can be fully integrated into the current drug development practices and improve drug development efficiency for the post-pandemic era.

## 1. Introduction

Drug development is a complex, lengthy, costly process. It takes more than 10 years from the discovery of a drug candidate for it to attain regulatory approval. Furthermore, the average cost of developing a new drug is 1.3 billion US dollars [1]. However, in a global health crisis such as the coronavirus disease 2019 (COVID-19) pandemic, in which treatments and preventive vaccines must be developed rapidly and efficiently to avert the pandemic, mounting costs and lengthy processes are certainly not welcomed.

The response to the COVID-19 pandemic at an unprecedented global scale has started shifting the old paradigm of drug development to improve the efficiency and productivity of drug research and development in the post-pandemic era. For example, ‘telehealth’ or non-face-to-face health care practices, involving the heavy use of digital technology, has received much attention [2]. Furthermore, scientists have applied artificial intelligence (AI) technologies to screening drug candidates to increase the success rate of the drug repositioning strategy. Engineers have also invented data tracking tools and data hubs to collect and share the development status of drug candidates and to update the results of clinical trials [3]. In addition, more and more public–private collaborations, many of which have occurred across the nations and enterprises, have been actively pursued. Furthermore, partly in response to the unavoidable challenges and changes in drug development, regulatory agencies such as the US Food and Drug Administration (FDA) and the European Medicines Agency (EMA) have tried to operate on a more flexible basis, while not undermining the scientific foundation of the drug review process.

The objectives of this review were threefold. First, we describe how the COVID-19 pandemic has disrupted the previous paradigm of drug development. Second, we anticipate how those disruptions could further reshape and transform drug development practices in the future, particularly for clinical development. Lastly, we discuss the issues and challenges in this transition, and how those roadblocks could be overcome.

## 2. Wide Acceptance of Telehealth

‘Telehealth’ is a type of health care in which information and communication technologies (ICT) play an indispensable role in delivering medical services to patients [4]. Telehealth is a complex term that covers a broad range of specialties and health-related services. Virtual visits and remote video monitoring are examples of telehealth [4].

Telehealth can minimize the possibility of transmitting infections between patients and health care providers. Therefore, the COVID-19 pandemic has hugely encouraged the adoption of telehealth. Compared with pre-COVID-19, health care providers are now seeing 50 to 175 times the number of patients via telehealth [2]. Clinicians have used telehealth to screen patients for COVID-19 using a heat detection device [2]. Likewise, doctors have monitored patients remotely and provided medical advice for quarantined patients or patients who are located in isolated areas, where access to medical services is limited [2,5].

Traditionally, clinical trials were performed at clinics and hospitals, thereby mandating patient visits and face-to-face on-site encounters between patients and healthcare providers. However, the COVID-19 pandemic has prompted the extensive adoption of decentralized clinical trials (DCTs), which are defined as clinical trials executed through telehealth technologies such as biosensors, wireless communication systems, and remote video monitoring [6,7]. For example, in a clinical trial to evaluate the efficacy and the safety of hydroxychloroquine to treat patients with COVID-19 infection (NCT04308668), participants and clinicians communicated via e-mails or text messages, without on-site visits [6]. This study also used commercial couriers to deliver the investigational drugs, i.e., hydroxychloroquine or placebos, to the study participants [6]. Patients’ visits to clinics can be minimized in DCTs using telehealth technologies. Thus, DCTs can reduce the patient’s burden in scheduling and traveling for clinic visits, and have the potential to improve patient retention rates in clinical trials, which eventually helps in patient recruitment. Likewise, DCTs using telehealth technologies can overcome geographical obstacles, expanding the access to clinical trials on a global scale, which was impractical and cumbersome, if not impossible, in traditional clinical trials.

Despite these benefits, telehealth may not be appropriate for all types of clinical trials [8]. For example, early-phase clinical trials to find the maximum tolerated dose of a drug candidate require frequent interventions, such as dose modifications, carried out by closely monitoring study participants, preferably in a confined area. Traditional designs are better suited for early-phase clinical trials because they need staffing capabilities and centralized resources. On the other hand, the lack of adequate ICT infrastructure may make it difficult to handle massive amounts of data coming from various telehealth devices [4,9]. Moreover, the lack of legislation and reimbursement mechanisms specific to telehealth are additional challenges in the wider use of telehealth [10,11]. To ensure that telehealth-based clinical trials are practical, safe, and efficient, both drug developers and engineers should actively validate telehealth technologies and publicly report their findings. The pharmaceutical industry has also raised concerns about unclear regulatory acceptance, noting that regulators are not fully ready to accept clinical endpoints reported mainly through telehealth devices. For example, twelve countries in the Organization for Economic Co-operation and Development (OECD) still have no national legislation on how to implement and manage telehealth services, although they have legalized the use of telehealth [9]. Because telehealth is a complex term requiring a wide range of specialties [4,9], securing a cross-disciplinary team, which consists of clinicians, health care providers, policy makers, and engineers of telehealth technologies, is crucial in developing standardized legislation specific to telehealth.

## 3. Drug Repositioning Revisited

Drug repositioning helps identify new therapeutic uses of an investigational or approved drug [12]. Drug repositioning could save time and expenses in drug development because the safety of repositioned drugs has already been tested in preclinical studies or clinical trials, though not completely [12]. In the COVID-19 pandemic, in which time is of the essence, drug repositioning has received attention in order to develop treatments and vaccines against SARS-CoV-2 at a much faster rate than before.

Typical drug repositioning consists of three steps: identifying repositionable candidates, assessing their effects in preclinical models, and evaluating their efficacy and safety in clinical trials. Traditionally, retrospective pharmacological analysis, performed mostly in a haphazard and non-systematic manner, has been used to identify repositionable candidates [13]. For example, sildenafil citrate was unexpectedly found to be effective for erectile dysfunction in clinical trials evaluating the efficacy of sildenafil as an angina treatment [13]. Furthermore, repositioning of thalidomide for erythema nodosum leprosum and multiple myeloma [14], aspirin for colorectal cancer [15], and raloxifene for breast cancer relied on empirical clinical experience and/or pharmacological analyses [16]. Likewise, in the early phase of the COVID-19 pandemic, several antiviral drugs such as remdesivir [17,18,19], chloroquine [20,21,22], and lopinavir/ritonavir [23,24], which showed broad-spectrum antiviral activity in several preclinical or clinical studies, had been repositioned for COVID-19 treatment [25]. However, few of those drugs repositioned against SARS-CoV-2 have shown satisfactory clinical efficacy [25].

To overcome the pitfalls of trial-and-error drug repositioning, researchers have searched for other approaches, of which AI is an example (Table 1) [13,26,27,28,29,30,31,32]. For instance, researchers from the US and Korea invented the Molecule Transformer-Drug Target Interaction deep learning model to identify repositionable antiviral candidates against SARS-CoV-2 [33]. Furthermore, to confirm the performance of the model, researchers compared the binding affinities of repositioned drugs against SARS-CoV-2 with FDA-approved drugs using AutoDock Vina, which is a widely used software for 3D-structure based docking and virtual screening [33]. Moreover, researchers and engineers from the Technical University of Munich have developed CoVex, a network medicine online platform that integrates published data about virus–human protein interactions, human protein–protein interactions and drug–target interactions for SARS-CoV-2 into a large-scale interactome [34]. CoVex platform users can systemically identify repositionable drugs against SARS-CoV-2 by mining the integrated virus–host–drug interactome [34,35]. In addition, the developers of CoVex have planned to extend the platform to other viruses, including influenza, Dengue fever, MERS, and Zika [34]. Likewise, an AI platform established by the National Health Research Institute in Taiwan identified eighty repositionable approved drugs targeting SARS-CoV-2. Among them, eight drugs (i.e., vismodegib, gemcitabine, clofazimine, celecoxib, brequinar, conivaptan, bedaquiline, and tolcapone) showed antiviral activities against the feline coronavirus in an in vitro cell-based assay [36]. Furthermore, using its AI platform and biomedical knowledge graph, BenevolentAI, a global company involved in the development and application of AI and computational medicine technologies, identified baricitinib, an oral Janus kinase (JAK) inhibitor, as a promising repositionable treatment against SARS-CoV-2 [30,31,32]. Eli Lily, who owns baricitinib, launched the Adaptive COVID-19 Treatment Trial (ACTT-2, *n* = *1034*, NCT04401579), in which the combination of baricitinib and remdesivir was found to reduce recovery time and progression to ventilation or death compared to remdesivir alone in COVID-19 patients in clinical status [37,38]. Based on the results of ACTT-2, on 19 November, 2020, the US FDA granted an Emergency Use Authorization (EUA) for barcitinib in combination with remdesivir, for the treatment of hospitalized COVID-19 adults and pediatric patients (2 years of age or older), who require invasive mechanical ventilation or extracorporeal membrane oxygenation (ECMO) [39]. Although computational-technology-aided drug repositioning strategies are in the nascent stage, this approach has shown the potential to be developed into a promising alternative to the current empirical drug repositioning methodology [25,40].

## 4. Real-Time Information Sharing: Data Tracking Tools and Hubs

As the severity of the COVID-19 pandemic has escalated, studies to evaluate the efficacy and safety of treatments and vaccines against severe acute respiratory syndrome coronavirus 2 (SARS-CoV-2) are being conducted at an unparalleled speed. For example, as of 19 March 2021, a total of 2803 clinical trials of COVID-19 treatments are being conducted worldwide [42]. Given the rapid speed at which the results of those clinical trials are updated, engineers have created data tracking tools to enumerate and identify the development status of the drug candidates and to update the results of their clinical trials. For example, Cytel, a multinational statistical software developer, developed an AI-based tracking tool to aggregate clinical trial data related to COVID-19 [3,42,43]. The tool pulls data from ClinicalTrials.gov, the Chinese Clinical Trial Registry, the EU Clinical Trials Register, Clinical Research Information Service (South Korea), International Standard Randomised Controlled Trial Number (ISRCTN), the Iranian Registry of Clinical Trials, the Japan Primary Registries Network, and the German Clinical Trials Register [3]. Cytel also built a real-time dashboard, a web portal that provides the overview of the global clinical trials of COVID-19 [42]. In addition, medical journals such as The New England Journal of Medicine [44], The BMJ [45], and The Lancet [46], have provided a data hub that shares trial reports, latest news, practical guidelines, and commentary about the COVID-19 pandemic. On the other hand, the state government of Georgia, US, has daily updated and provided geospatial analyses (e.g., Georgia’s COVID-19 case summary, death data summary, and maps of regional hospital capacity) via its COVID-19 data hub, using data pooled from labs, hospitals, and health care providers [47]. Data tracking tools and data hubs have provided researchers with various COVID-19-specific forms of content, especially timely information and updates regarding clinical trials. This has eventually allowed researchers to avoid duplicating efforts and expenses by screening out existing or active clinical research [3].

## 5. Public–Private Collaborations Expanded

Historically, drug development has been led by distinct players, with minimal collaboration between them. Biopharmaceutical companies, academia, and the National Institutes of Health (NIH) in the US or similar government-funded research organizations in other countries have worked independently with different goals, processes, and success criteria [48]. Likewise, individual pharmaceutical companies competed with each other to gain the upper hand in the drug market. Therefore, little to no collaboration was successfully pursued in the old era of drug development.

However, the COVID-19 pandemic has changed the situation. In the midst of the pandemic, academia, nonprofit organizations, governments, and biopharmaceutical companies avidly opened up collaborations with each other to address the public health crisis at a global scale. For example, the US NIH established ‘Accelerating COVID-19 Therapeutic Interventions and Vaccines (ACTIV)’, a public–private partnership, which consists of academia, government agencies (e.g., the US FDA), non-profit organizations (e.g., the Bill and Melinda Gates Foundation), and industry (i.e., twenty pharmaceutical companies) [49]. ACTIV aims to accelerate COVID-19 drug development by efficiently utilizing limited biomedical resources and prioritizing the most promising vaccine candidates against SARS-CoV-2 [49]. Moreover, governments around the world have joined the COVAX Facility, a global risk-sharing mechanism for COVID-19 vaccines [50]. The COVAX Facility, co-led by the World Health Organization (WHO), the Infectious Diseases Innovation Association (CEPI), and the World Vaccine Immunity Association (GAVI), aims to provide an equal supply of vaccines to at least 20% of the world’s population [50].

Biopharmaceutical companies have also actively formed partnerships between themselves, many of which have occurred across nations and enterprises (Table 2). For example, CSL Behring and Takeda co-founded the ‘CoVIg-19 Plasma Alliance’, which includes leading global plasma companies (i.e., Biopharm Plasma, Biotest, GC Pharma, Octapharma, LFB, NBI, and Sanquin) [51]. The CoVIg-19 Plasma Alliance, supported by the National Institute of Allergy and Infectious Diseases (NIAID), NIH, US, has been developing hyperimmune globulin (H-Ig), a non-branded plasma-derived medicine for COVID-19 [51]. Furthermore, a French biopharmaceutical company, Sanofi, and an English biopharmaceutical company, GlaxoSmithKline (GSK), combined their innovative technologies to effectively develop a COVID-19 vaccine [52]. Based on Sanofi’s DNA recombination technology, Sanofi has contributed to producing the spike protein of SARS-CoV-2 [52]. On the other hand, GSK, which has a vaccine portfolio of more than twenty diseases [53], has contributed its proven adjuvant technology to reduce the amount of vaccine protein required per dose [52]. Furthermore, the American pharmaceutical company Pfizer and the German biotechnology company BioNTech co-developed BNT162, an mRNA COVID-19 vaccine [54]. Likewise, Moderna, a Boston-based company, developed an mRNA-1273 vaccine in collaboration with the NIAID, NIH, US [55]. In addition, AstraZeneca, a British–Swedish pharmaceutical company, collaborated with the University of Oxford to advance basic vaccinology research [56]. Through collaboration, pharmaceutical companies have been able to access external know-how and knowledge and have also been able to maximize the returns on their research and development. As a result of these monumental and innovative collaborations, vaccines against SARS-CoV-2 are being developed at an unrivalled pace [57]. As of 16 April, 2021, a total of 13 COVID-19 vaccine candidates have received regulatory approvals in the world for full or limited use (Table 3) [57,58].

Because vaccine manufacturing under good manufacturing practices (GMPs) is a highly complex process and requires substantial financial investments [59], various partnerships between vaccine developers and manufacturers have been established to enable the scaled-up production of those COVID-19 vaccines under GMPs [60]. For example, South Korea’s biopharmaceutical company SK Bioscience signed a contract manufacturing organization (CMO) deal and a contract development and manufacturing organization (CDMO) deal with AstraZeneca [61] and Novavax [62], respectively. SK Bioscience will produce up to 500 million doses of AstraZeneca’s COVID-19 vaccine and 40 million doses of Novavax’s COVID-19 vaccine [61,63]. Likewise, Moderna has made a 10-year strategic agreement with Lonza, a Swiss multinational chemicals and biotechnology company [64]. The purpose of the agreement was to enable the manufacturing of up to 1 billion doses of mRNA-1273 per year, as well as to prepared for the future manufacturing of additional products in Moderna’s extensive clinical portfolio [64].

The COVID-19 pandemic has prompted various types of collaborations to increase the efficiency of drug development and to avert the global pandemic. The present is the most opportune time to set aside individualism and to adopt open approaches more actively for ground-breaking drug research and development.

## 6. Changes and Challenges in the Regulatory Agencies

Historically, strict regulations, inflexible procedures, and time-consuming review processes by regulatory agencies have been criticized for delayed marketing approvals of drugs [65]. However, the COVID-19 pandemic has prompted the regulatory agencies to operate on more simplified and flexible administrative procedures and review bases than before, while not undermining the scientific foundation of drug review.

Many regulatory agencies have established emergency taskforces or programs to support drug developers and to take prompt regulatory actions [66,67]. For example, the EMA has established the COVID-19 EMA pandemic taskforce (COVID-ETF) and the EMA COVID-19 steering group to preemptively address possible delays in the review of COVID-19 treatments and vaccines, as well as for non-COVID-19-related assessments [67]. COVID-ETF provides scientific support and feedback for drug developers to expedite clinical trials and development plans for COVID-19 medicines [67,68]. The EMA COVID-19 steering group has not only coordinated the activities of COVID-ETF, but has also revised the procedures to introduce more flexibility for the fast-tracked approval of medicines [67]. Similarly, the FDA has accepted data from diverse sources in regulatory decision-making to combat the COVID-19 pandemic, even if the data were not obtained from clinical trials. In June 2020, the FDA, in a collaboration with the NIH, formed the CURE Drug Repurposing Collaboratory (CDRC), a partnership that initiated the COVID-19 pilot program to collect real-world data for identifying potential treatments for SARS-CoV-2 [66]. CDRC has utilized real-world data to support and power randomized clinical trials in which it was difficult to enroll a sufficient number of COVID-19 patients [66,69].

Thirteen years ago, PricewaterhouseCoopers (PwC), a multinational management consulting firm, anticipated that by 2020 regulators would grant limited marketing approval based on ‘live licenses’, which are conditional approvals in a restricted patient populations and/or with a narrow indication for further in-life-testing to evaluate long-term safety and efficacy in various populations [70]. These predictions have proved to be correct, as the US FDA and the EMA have granted many EUAs to address the need for diagnostic kits, treatments, and vaccines for COVID-19 in a timely manner. EUAs do not constitute full regulatory approval, which typically requires the submission of substantial evidence for the safety and efficacy of a drug, diagnostic kit, or medical device [39]. In a public health crisis such as the COVID-19 pandemic, however, the regulatory agencies had to balance the unquestionable long-term safety and efficacy of the vaccines or treatments and the urgent need for them by the public. Stephen M. Hahn, the US FDA Commissioner, has stressed that, in a situation where the two demands conflict, the regulatory agencies should consider granting an EUA if the risks of not having treatments or vaccines are much greater than the risks associated with the products themselves [71].

However, the public has raised concerns about the scientific rigor and transparency of the EUA process. On 28 March, 2020, without disclosing the evidence underlying the decision, the US FDA granted an EUA for the use of hydroxychloroquine for the treatment of COVID-19 [71,72]. Not long afterwards, on 15 June, 2020, the US FDA revoked an EUA of hydroxychloroquine, noting that hydroxychloroquine was not effective in reducing mortality or in speeding up the recovery of COVID-19 patients [73]. Such erroneous and poor decision-making has reduced the credibility and public confidence in regulatory agencies’ decisions [74]. Kyle Thomson, the FDA’s Chief Counsel from 2012 to 2020, emphasized that regulatory agencies should clarify evidence standards for EUAs [71]. Additional clarity and transparency in EUA standards and processes will increase the consistency and quality of regulatory agencies’ decisions on EUAs, thereby increasing the trust and confidence of the public [71,74].

## 7. Conclusions

The COVID-19 pandemic has encouraged the adoption of disruptive approaches in various areas of drug development. For example, telehealth, strengthened by the heavy use of digital technologies and data tracking tools that enable real-time information sharing, have increased the efficiency and practicality of clinical trials in terms of trial conduct and patient enrollment. Likewise, computational breakthroughs have increased the success rate of drug repositioning strategies. Furthermore, drug developers have actively adopted open innovation approaches, which are expected to increase the efficiency and productivity of drug development, and regulatory agencies have attempted to operate with more simplified and flexible administrative procedures and review methodologies than before. However, it is unclear whether these disruptions will appreciably improve the efficiency of drug development compared with conventional methods. Certainly, these disruptions could be short-lived as the public health threats by the COVID-19 pandemic vanish. Therefore, to maintain the positive effects of these disruptions, a collaborative community of experts should constantly strive to figure out how these disruptive technologies can be integrated into current drug development practices. This will, in turn, stimulate the paradigm shift in drug discovery and development created during the COVID-19 pandemic.

## Figures and Tables

**Table 1:** Computational-technology-aided drug repositioning strategies used to identify repositionable drugs for SARS-CoV-2.

Methods or Models	Data or Software Used	Identified Repositionable Drugs for SARS-CoV-2	References
MT-DTI Deep Learning Model	NCBI Database, DTC Database, BindingDB Database, DrugBank Database (SMILES)	Atazanavir, Remdesivir, Kaletra, Rapamycin, Tiotropium Bromide	[33]
Deep Neural Network Model	DrugBank Database (Data of Approved Drugs and 3C-Like Protease Inhibitors)	Bedaquiline, Brequinar, Celecoxib, Clofazimine, Conivaptan, Gemcitabine, Tolcapone, Vismodegib	[36]
Pharmacology-Based Network Model	NCBI GenBank Database, EMBL-EBI database, DrugBank Database (SMILES), Therapeutic Target Database, PharmGKB Database, ChEMBL, BindingDB, IUPHAR/BPS Guide to PHARMACOLOGY90, UniProt Database	Irbesartan, Toremifene, Camphor, Equilin, Mesalazine, Mercaptopurine, Paroxetine, Sirolimus, Carvedilol, Colchicine, Dactinomycin, Melatonin, Quinacrine, Eplerenone, Emodin, Oxymetholone	[28]
Hierarchical Virtual Screening (MMFF-Based Free Energy Calculation Methods)	Schrodinger Software, OpenBabel Software, AMBER Software (Molecular Dynamics Simulation), DrugBank Database (DTIs)	Carfilzomib, Eravacycline, Valrubicin, Lopinavir, Elbasvir, Streptomycin	[29]
BenevolentAI Platform (MCTS algorithm and Deep Neural Network Model)	Reaxys Chemistry Database, ZINC Database	Baracitinib, Fedratinib, Sunitinib, Erlotinib	[30,31,32]
Physics-Based Glide Algorithm	Schrodinger Software, Broad Repurposing Library, Biotek Gen5 Software, GraphPad Prism 8	Boceprevir, Ciluprevir, Narlaprevir, Telaprevir	[41]

SARS-CoV-2, severe acute respiratory syndrome coronavirus 2; MT-DTI, molecule transformer–drug target interaction; NCBI, National Center for Biotechnology Information; DTC, Drug Target Commons; SMILES, Simplified Molecular-Input Line-Entry System; AI, artificial intelligence; MMFF, molecular mechanical force field; DTI, drug–drug target interaction; MCTS, Monte Carlo tree search.

**Table 2:** COVID-19 vaccines and partnerships [57,58].

Product	Developer	In Partnership with
Ad26.COV2.S	Johnson & Johnson	Beth Israel Deaconess Medical Center
AG0302-COVID19	AnGes	Osaka University and Takara Bio
ARCoV	Academy of Military Medical Sciences	Suzhou Abogen Biosciences and Walvax Biotechnology
Ad5 and Ad35	Cellid	LG Chem
Comirnaty	Pfizer	BioNTech
Convidecia	CanSino Biologics	Academy of Military Medical Sciences
Covaxin	Bharat Biotech	Indian Council of Medical Research and the National Institute of Virology
COVID-19 viral protein	Sanofi	GSK
CoVLP	Medicago	GSK
ChulaCov19	Chulalongkorn University	Chula Vaccine Research Center
DS-5670	Daiichi Sankyo	University of Tokyo
GBP510	University of Washington	SK Bioscience and GSK
GRAd-COV2	ReiThera	Lazzaro Spallanzani National Institute for Infectious Diseases
HGC019	Gennova Biopharmaceuticals	HDT Bio
mRNA-1273	Moderna	NIH
mRNA Vaccine	Arcturus Therapeutics	Duke-NUS Medical School
S Protein of COVID-19	Clover Biopharmaceuticals	Dynavax.
Vaxzevria	University of Oxford	AstraZeneca
ZF2001	Anhui Zhifei Longcom	The Institute of Medical Biology at the Chinese Academy of Medical Sciences

Updated 16 April, 2021. Data from The New York Times, based on reports from state and local health agencies. COVID-19, coronavirus disease 2019; NIH, National Institutes of Health; GSK, GalxoSmithKline.

**Table 3:** Current approval status of COVID-19 vaccines [57,58].

Developer	Product	Approved for Full Use in	Approved for Emergency or Early Use in
Pfizer and BioNTech	Comirnaty	Bahrain, Brazil, New Zealand, Saudi Arabia, Switzerland	US, EU, UK, Argentina, Australia, Botswana, Canada, Costa Rica, Greenland, Hong Kong, Iceland, Iraq, Japan, Kuwait, Lebanon, Mexico, Norway, Panama, Peru, South Africa, South Korea, Thailand, Turkey, UAE, other countries
Moderna	mRNA-1273	Switzerland	US, EU, UK, Canada, Greenland, Guatemala, Iceland, Israel, Mongolia, Norway, Qatar, Singapore, Thailand, Vietnam
Johnson & Johnson	Ad26.COV2.S	Not Approved	US, EU, Brazil, Canada, Colombia, Greenland, Iceland, Liechtenstein, Norway, South Africa, South Korea, Switzerland, Thailand
Oxford and AstraZeneca	Vaxzevria	Brazil	EU, UK, Algeria, Argentina, Australia, Bahamas, Brazil, Brunei, Canada, Chile, Colombia, Dominican Republic, Egypt, EI Slavador, Greenland, Hungary, Iceland, Mexico, Namibia, Sri Lanka, South Africa, South Korea, Vietnam, other countries
**Gamaleya Research Institute**	Sputnik V	Not Approved	Russia, Algeria, Argentina, Bahrain, Bosnian Serb Republic, Cameroon, Congo Republic, Djibouti, Egypt, Hungary, Honduras, Iran, Iraq, Jordan, Laos, Lebanon, Mali, Morocco, North Macedonia, Paraguay, Palestinian Authority, Philippines, Sri Lanka, UAE, other countries
Sinovac	CoronaVac	China	Azerbaijan, Brazil, Cambodia, Chile, Colombia, Ecuador, Hong Kong, Indonesia, Laos, Malaysia, Mexico, Pakistan, Panama, Philippines, Thailand, Tunisia, Turkey, Ukraine, Uruguay, Zimbabwe
Sinopharm (Wuhan)	Vero Cells	China	UAE
Sinopharm (Beijing)	BBIBP-CorV	Bahrain, China, UAE	Argentina, Brunei, Cambodia, Egypt, Gabon, Guyana, Hungary, Iran, Iraq, Jordan, Maldives, Namibia, Nepal, Pakistan, Peru, Venezuela, Zimbabwe
FBRI	EpiVacCorona	Turkmenistan	Russia
Chumakov Center	KoviVac	Not Approved	Russia
CanSino Biologics	Convidecia	China	Chile, Hungary, Mexico, Pakistan
Bharat Biotech	Covaxin	Not Approved	India
Anhui Zhifei Longcom	ZF2001	Not Approved	China, Uzbekistan

Updated 16 April, 2021. Data from The New York Times, based on reports from state and local health agencies. COVID-19, coronavirus disease 2019; US, United States; EU, European Union; UK, United Kingdom; UAE, United Arab Emirates.

## Data Availability

Not applicable.
